# The angiogenic asset of soft tissue sarcomas: a new tool to discover new therapeutic targets

**DOI:** 10.1042/BSR20140075

**Published:** 2014-11-04

**Authors:** Laura Rocchi, Stefano Caraffi, Roberto Perris, Domenica Mangieri

**Affiliations:** *Unità Operativa di Anatomia e Istologia Patologica, Azienda Ospedaliero-Universitaria di Parma, Via Gramsci, 14, 43100-Parma, Italy; †COMT–Centro di Oncologia Medica e Traslazionale, Università di Parma, Parco Area delle Scienze 11/A 43100-Parma, Italy

**Keywords:** angiogenesis factors, angiogenesis, soft tissue sarcomas, target therapy, CSF, colony-stimulating factor, EC, endothelial cell, FGF-2, fibroblast growth factor-2, MFH, malignant fibrous histiocytoma, MMP, matrix metalloproteinase, mTOR, mammalian target of rapamycin, MVD, microvessels density, PDGFRβ, platelet-derived growth factor beta, PlGF, placental growth factor, STS, soft tissue sarcomas, TKI, tyrosine kinase inhibitor, TIMP, tissue inhibitors of metalloproteinases, uPa, urokinase-type plasminogen activator, VEGF, vascular endothelial growth factor, VEGFR, VEGF receptor, vWf, von-Willebrand factor

## Abstract

STS (soft tissue sarcomas) are rare malignant tumours deriving from cells of mesenchymal origin and represent only 1% of all malignant neoplasms. It has been extensively demonstrated that angiogenesis has an important role in cancer malignancy. Particularly, a lot of studies demonstrate the importance of angiogenesis in the development of carcinomas, whereas little is known about the role of angiogenesis in sarcomas and especially in STS. This review aims at summarizing the new discoveries about the nature and the importance of angiogenesis in STS and the new possible therapeutic strategies involved. Only a few studies concerning STS focus on tumour neovascularization and proangiogenic factors and look for a correlation with the patients prognosis/survival. These studies demonstrate that intratumoural MVD (microvessels density) may not accurately represent the angiogenic capacity of STS. Nevertheless, this does not exclude the possibility that angiogenesis could be important in STS. The importance of neoangiogenesis in soft tissue tumours is confirmed by the arising number of publications comparing angiogenesis mediators with clinical features of patients with STS. The efficacy of anti-angiogenic therapies in other types of cancer is well documented. The understanding of the involvement of the angiogenic process in STS, together with the necessity to improve the therapy for this often mortal condition, prompted the exploration of anti-tumour compounds targeting this pathway. In conclusion, this review emphasizes the importance to better understand the mechanisms of angiogenesis in STS in order to subsequently design-specific target therapies for this group of poorly responding tumours.

## Abstract 2

Angiogenesis is important for tumour growth and metastatization. The better understanding of the mechanisms of angiogenesis in soft tissue sarcomas can lead the design of specific target therapies for this group of poorly responding tumours.

## INTRODUCTION

STS (soft tissue sarcomas) are rare malignant tumours deriving from cells of mesenchymal origin and represent only 1% of all malignant neoplasms. They can occur everywhere in the body but they preferentially develop in the extremities, the trunk, the retroperitoneum or the head and neck [1]. Among more than 50 histological types of STS known, the most common are malignant fibrous histiocytoma, leiomyosarcoma, liposarcoma, synovial sarcoma, fibrosarcoma and malignant peripheral nerve sheath tumours [1]. It has been extensively demonstrated that the growth and metastatic abilities of solid tumours depend on angiogenesis. Angiogenesis is defined as the development of new blood vessels from a pre-existing vascular bed. This process is tightly controlled by a balance in the promoters and inhibitors of angiogenesis, which is altered in various disease states. In tumours, an excessive spread of new vessels promotes growth and metastasis [2].

### STS angiogenic asset

In many tumours, neovascularization correlates with the disease stage and can have prognostic significance. Particularly, a lot of published reports have shown a significant correlation between intratumoural MVD (microvessels density) and the metastatic disease and/or patient survival in different kind of carcinomas such as breast carcinomas, gastrointestinal carcinoma, melanoma, prostate carcinoma, testicular carcinoma, ovarian carcinoma, bladder carcinoma, central nervous system tumours, multiple myeloma, non-small cell lung carcinoma and squamous cell carcinoma of the head and neck. Only a few studies of this kind are reported for STS. In an early study by Oshawa et al. [3], MVD was examined in 42 cases of MFH (malignant fibrous histiocytoma). Microvessels were identified by anti-FVIII-RA (factor-VIII-related antigen)-antibody and counted, but no correlation between their number and metastasis was found, and the authors concluded that angiogenesis is ‘apparently’ not a key factor. The result was then confirmed in other reports. Saentz et al. [4] investigated the significance of tumour angiogenesis in 119 primary, high-grade extremity STS. Angiogenesis measured by factor VIII staining had no prognostic significance in these types of soft tissue tumours, too. In another study, 54 primary and recurrent synovial sarcomas also showed no correlation between MVD and prognosis [5]. Similar results were obtained for leiomyosarcoma and MFH [6]: using an anti-vWf (von-Willebrand factor) staining to identify the microvessels, the authors confirmed what had been proposed in the previous studies. Nevertheless, the authors compared the microvessel pattern between leiomyosarcomas and breast carcinomas, identifying some specific characteristics: in the analysed STS the capillaries were homogeneously distributed, while in the carcinomas they tended to form clusters in the central areas of the tumour body. West et al. [7], using a different marker (CD31) to stain the microvessels, demonstrated that MVD alone does not predict metastasis of the primary tumour, and that the peripheral edge of the tumour has the lowest mean MVD, followed by the centre of the mass and then by the necrotic areas. These two studies focused the attention on the importance of the distribution of the microvessels rather than their density. In our laboratory, we have observed aberrant vascularization and pericyte coverage, defined via anti-CD31 and anti-PDGFRβ (platelet-derived growth factor beta) receptor antibodies, respectively, in various leiomyosarcomas (unpublished work). Overall, these data reveal that only a limited number of studies concerns the immunohistological assessment of microvasculature in STS. The use of different kinds of antibodies directed to different vessels marker to identify the microvasculature in STS needs to be discussed. Neoangiogenesis has an important role in tumour growth and metastatization but the often used anti-factor-VIII-related antigen and anti-vWf do not discriminate between tumour neoangiogenesis and preexisting vasculature. Indeed, the use of an anti-CD34 antibody does not permit to distinguish between the blood and the lymph vessels and thus renders evaluating the microvascular patterns and the rate of neoangiogenesis really difficult. That is probably the reason why MVD may not accurately represent the angiogenic capacity of STS. This does not exclude the possibility that angiogenesis could be important in STS as much as in carcinomas as discuss below.

### Involvement of angiogenesis factors in STS

The importance of neoangiogenesis in soft tissue tumours is confirmed by the arising number of publications comparing angiogenesis mediators [VEGF (vascular endothelial growth factor), angiogenin, etc.] with clinical features (tumour grade, tendency to metastasize, response to treatment, overall survival and risk of recurrence) of patients with STS. For an overview of the different angiogenic factors and a correlation with prognosis in STS where available refer to Table 1.

**Table 1:** Overview of different angiogenic factors and their correlation with STS prognosis The table summarizes the principal angiogenic factors and their correlation, were reported, with STS prognosis (+). Prognosis information missed or incomplete are indicated with -. VEGF, vascular endothelial growth factor; FGF, fibroblast growth factor; PDGF, platelet derived growth factor; PlGF, placenta growth factor; IGF, insulin-like growth factor; Ang, angiopoietin; EGF, epidermal growth factor; HGF, hepatocyte growth factor; HIF, hypoxia-inducible factor; TGF, transforming growth factor; TNF, tumour necrosis factor; IL, interleukin; NP, neuropilin; SDF, stromal cell-derived factor; TSP, thrombospondin; PF, platelet factor; TIMP, tissue inhibitor metalloproteinase.

Category	Major functions	Names	Correlation with prognosis	Reference(s)
Angiogenesis inducers	Induction of EC (endothelial cell) growth, proliferation and survival under stress; induction of migration of ECs during sprouting angiogenesis; increase vascular permeability of ECs; stimulate secretion of proteinases important for tumour invasion and progression; activate matrix degrading enzymes (e.g. metalloproteinases); recruitment of tissue infiltrating hematopoietic cells, modulates receptor-ligand interaction. HIF-1α and angiogenin are transcriptional controller of many of the factors indicated	VEGF-A	+	[8,9,50]
		VEGF-B	+	[8,10]
		VEGF-C	+	[8,11]
		VEGF-D	−	[8]
		FGF-1	−	[12]
		FGF-2	+	[13,55]
		PDGF	+	[9,14,15,55]
		PlGF	+	[8,16]
		IGF-I/II	−	[17]
		Ang-1	−	[18]
		EGF	+	[11,19]
		HGF	+	[20,21]
		HIF-1α	+	[11,22]
		TGF-α	−	[23]
		TGF-β	+	[24,25]
		TNF-α	−	[26]
		IL-1	−	[27]
		IL-8	+	[28]
		NP1/2	−	[29]
		Angiogenin	−	[30]
		SDF-1	−	[31]
Angiogenesis inhibitors	Inhibition of ECs proliferation and growth and induction of ECs apoptosis; inhibit ion of ECs migration and blood vessels maturation; inhibition of the metalloproteinases activity or uPa activity	TSP-1/2	−	[32,33]
		Angiostatin	−	[34]
		Endostatin	+	[35,36]
		Vasostatin	−	[37]
		PF-4	−	[38]
		Antiangiogenic Antithrombin III	−	[39]
		TIMPs	+	[40–43]
		Ang-2	−	[44,45]

VEGF-A is the best-studied angiogenesis mediator. In different studies, a positive correlation was assessed by immunohistochemistry [46,47] and ELISA-based methods [48,49]. These works demonstrate that high levels of VEGF-A in tumours and blood samples from STS patients are associated with higher tumour grade, increased tendency to form metastasis, reduced response to treatment, lower overall survival and increased risk of recurrence. In a most recent study [50], tissue microarrays of STS from 249 patients were used to investigate the prognostic impact of all VEGF and VEGFRs (VEGF receptors). The authors confirmed what was previously reported for VEGF-A and its receptors, VEGFR-1 and 2 and, more importantly, they reported for the first time VEGFR-3 expression as an independent negative prognostic marker. Recent data have shown that VEGFR-3, traditionally associated with the lymphatic endothelium, is also expressed in the lamellipodia of lead-cells in angiogenic sprouts, indicating that VEGFR-3 may play an important role in blood vessel angiogenesis [51]. Although these results might seem to contrast with the conclusions obtained by MVD analysis, this apparent incongruity may reflect the above-mentioned methodological difficulties in determining the accurate count of newly formed microvessels, or the possibility that VEGF may play different roles in tumour growth.

Other proangiogenic factors are up-regulated in STS. PDGFβ expression, measured by quantification of both protein and mRNA, is significantly tied with tumour grade and cell proliferation in soft-tissue sarcomas [6,52]. High levels of circulating angiopoietin-2 and FGF-2 (fibroblast growth factor-2) are also found in patients with STS in respect with healthy controls [53–55]. Recently, Kilvaer et al. concluded that FGF-2, alone or in co-expression with PDGF-B and VEGFR-3, is a significant negative prognostic factor in resected STS patients [55]. Other important mediators indirectly involved in neoangiogenesis are the MMPs (matrix metalloproteinases), particularly MMP9 and MMP2, the uPa (urokinase-type plasminogen activator) and the TIMPs (tissue inhibitors of metalloproteinases), particularly TIMP2, all responsible for extra-cellular matrix degradation, another important step in tumours angiogenesis, invasion and metastasis. Increased levels of MMP2, MMP9 and uPa are associated with poor prognosis in different series of patients with STS [56,57]. Apart from the few data available on the status of vascularization in STS, all the studies about the quantification of proangiogenic factors mentioned indirectly demonstrate the importance of angiogenesis in STS. Some *in vivo* studies, using murine tumour models of STS, show clearly the consequences of an increased expression of various angiogenic factors. Murine T241 fibrosarcoma cell lines, engineered to stably overexpressed VEGF-A and -C, were implanted into immunodeficient mice to generate tumour xenografts. VEGF-A and -C expressing tumours displayed significantly accelerated growth compared with the non-VEGF expressing counterparts. At the same time, they showed a markedly increased tumour vessels density, although tumour vasculature was primitive and disorganized, with reduced pericytes association and improved vascular permeability [58]. In another murine model of fibrosarcoma [59], tumours proved to be very invasive *in vivo* and exhibited highly irregular vessels, variable in shape and diameter. Interestingly, only a few vessels had continuous CD31 staining whereas most of them showed gaps or even absence of CD31 reactivity. The same aberrations were shown for laminin staining, a marker of the basal membrane. In addition, VEGF-A was secreted at highly levels and a microarray analysis demonstrated an increased expression of MMP2.

In this context, it is reasonable to hypothesize that tumour cells by themselves, under the stimulus of the factors secreted, would contribute to the formation and delineation of the vascular channel structures and lacunae as had previously been proposed for other kinds of sarcoma [60,61]. In any case, disputing the involvement of angiogenic factors in STS, the principal mechanism proposed is that FGF-2 could recruit endothelial cells and increase the release of MMPs and uPa leading to extracellular matrix degradation and permitting tumour motility, vascular smooth muscle cells recruitment trough PDGF and pericytes coverage of newly formed vessels [55].

The molecular mechanisms through which pro/anti-angiogenic factors can influence angiogenesis in soft tissue tumour growth are not clearly understood. Generally it could be postulated that the main pro-angiogenic factors involved (VEGF, FGF-2 and PDGF), because of their nature of growth factors, act on the tumour cells in a paracrine/autocrine loop, activating intracellular pathways that drive cell proliferation, apoptosis abrogation and anti-growth signals [55]. One such intracellular pathway is the mTOR (mammalian target of rapamycin) pathway, a key regulator of protein translation. Some growth factors such as VEGF and PDGF can activate multiple pathways, including AKT, ERK, p38 and IKKβ that in turn converge on TSC1/2 activating mTOR, which may promote angiogenesis via control of the HIF (hypoxia inducible factor)-1α [62]. This process is well demonstrated for Kaposi's sarcoma [63,64] but could be active in STS too.

### Targeting the angiogenesis in STS

The understanding of the involvement of the angiogenic process in STS, together with the necessity to improve the therapy for this often mortal condition, prompted the exploration of antitumour compounds targeting this pathway. There are two drug classes by which the angiogenic pathway can be inhibited directly [monoclonal antibodies and TKIs (tyrosine kinase inhibitors)] and one class by which the angiogenic pathway can be inhibited indirectly (rapalogues). For a more comprehensive review of anti-angiogenic therapy in soft tissue sarcoma and their mode of action; see [65,66]. Below is given only a general panoramic of anti angiogenic drugs used in soft tissue therapy. All the compounds that have been trialed in soft tissue sarcoma or that are in clinical trial at present are summarized in Table 2.

**Table 2:** Antiangiogenic agents trialed in soft tissue sarcoma Antiangiogenic agents divided by their mode of action. Their targets are reported. In addition, the results of completed clinical trials or the presence of ongoing clinical trials are reported. (+) positive results, (−) negative results, (=) not properly clear results and (X) no results published. VEGF, vascular endothelial growth factor; PDGFR, platelet-derived growth factor receptor; IGF-1R, insulin growth factor receptor; DR, death receptor; VEGFR, vascular endothelial growth factor receptor; FGFR, fibroblast growth factor receptor; EGFR, epidermal growth factor receptor; FKBP12, FK506 binding protein; FGF, fibroblast growth factor; HGF, hepatocyte growth factor; IL, interleukin.

Mode of action	Drug	Target	Response	Comments	Reference(s)
Monoclonal antibody	Bevacizumab	VEGF	+	Bevacizumab in combination with other drugs; often the benefit of adding bevacizumab remains unclear	[66–70]
	IMC-3G3	PDGFRα	+	Only Phase I studies results available. A Phase Ib/II study is ongoing (NCT01185964)	[71]
	TRC105	CD105	X	A Phase 1B Dose-escalation Study of TRC105 in Combination With Pazopanib is ongoing (NCT0197551)	[72]
	Cixutumumab	IGF-1R	+		[73–77]
	Figitumumab	IGF-1R	+	Only Phase I studies results available	[78,79]
	AMG 479	IGF-1R	+	Open Label Extension Study (Phase II) of AMG 479 and AMG 479 (NCT01327612)	[80,81]
	AMG 655	DR 5	+	Open Label Extension Study (Phase II) of AMG 479 and AMG 479 (NCT01327612)	[82]
	Vitaxin	Integrin α_V_β_3_	=	Safe and potentially active Only few cases of soft tissue sarcoma analysed	[83–85]
Tyrosine kinase inhibitor	Sunitinib	VEGFR-1, VEGFR-2,VEGFR-3,PDGFR-α/β, KIT	+	Sunitinib demonstrated notable evidence of metabolic response in several patients with non-GIST sarcoma but a deeper subtypes stratification is needed	[86–89]
	Sorafenib	VEGFR-2,VEGFR-3,PDGFR,c-RAS, b-RAF,KIT	+		[90–93]
	Pazopanib	VEGFR-1, VEGFR-2,VEGFR-3,PDGFR-α/β, KIT	+		[94–97]
	Dasatinib	VEGFR-2, PDGFR, BCR/ABL, Src and KIT	X	Three active clinical trials (NCT00464620, NCT01643278 and NCT00788125	
	Brivanib	VEGF-R2 and FGF-R1 and -2	X	Clinical trial NCT00633789, completed	
	Cediranib	VEGFR-1,VEGFR-2, VEGFR-3	+	Single-agent activity on alveolar soft part sarcoma. A phase II trial (NCT01391962) is currently being conducted for patients with alveolar soft part sarcoma comparing cediranib with sunitinib	[98,99]
	Tivantinib	c-Met	X	Phase Ib Study of the Combination of Pazopanib, and Tivantinib, in Patients With Refractory Advanced Solid Tumours (NCT01468922)	[100]
	Axitinib	VEGFR-1,VEGFR-2,VEGFR-3,PDGFR and c-KIT	X	Phase II study (NCT01140737)	
	Semaxanib	VEGFR-2, KIT	−	No other ongoing clinical studies	[101]
	Cabozantinib	MET,VEGFR-2, RET	X	One Phase II ongoing clinical study (NCT01755195)	
	Saracatinib	src	X	One Phase II clinical trial completed (NCT00659360)	
	Gefitinib	EGFR, HER1	−	Gefitinib did not demonstrate sufficient activity	[102,103]
	Regorafenib	VEGFR and Tie-2	X	Three Phase II clinical trials ongoing (NCT01900743 NCT02048371 NCT02048722)	
Rapalogues (mTORC1 tyrosine kinase inhibitors)	Temsirolimus	FKBP12	+	Temsirolimus has limited clinical activity. The oucomes can be improved by a combinatorial therapy	[74,76,104,105]
	Sirolimus	FKBP12	−	Objective tumour response was infrequent	[106]
	Ridaforolimus	FKBP12	+		[107–109]
	Everolimus	FKBP12	+	Phase II clinical study in combination with Imatinib (NCT01281865) Phase II clinical study ongoing in Children and Adolescents (NCT01216839)	[78]
Other	ABT-510	VEGF, bFGF, HGF, IL8	+	Thrombospondin analogue; Strong single-agent activity need to be more clarify.	[110–112]
	Aflibercept (VEGF Trap)	VEGF	+	Soluble decoy receptor. Modest activity Active a Phase II clinical trial (NCT00390234)	[113,114]

Bevacizumab is the only anti-VEGF monoclonal antibody that has a proven activity against some forms of STS, especially in combination with other chemotherapic agents such as doxorubicin. With respect to TKIs, four drugs have been assessed for their effects against STSs: Sunitinib, active against VEGFR-1, 2, 3, PDGFR and KIT; Sorafenib, targeting VEGFR-2 and 3, PDGFR, Raf and KIT; Pazopanib, an inhibitor of VEGFR-1, -2 and -3, PDGFR and KIT; and the new Dasatinib, that blocks VEGFR-2, PDGFR and the src-family kinases.

Rapalogues or rapamycin analogues, are a group of four compounds acting on mTOR that are nowadays under investigation on STS. In general, most of these treatments do not increase the overall survival in the majority of the patients, but a significant tumour contraction and a stable disease are usually the best responses reported. New therapeutic strategies, involving the combination of known targeted therapies with conventional cytotoxic drugs and the targeting of other components involved in angiogenesis, need to be developed.

In this context, it is important to mention what was seen by Grabellus et al. [115]. In a series of 53 STS patients, isolated limb perfusion with TNFα (tumour necrosis factor alpha) and Melphalan (TM-ILP) was performed. Patients were highly responsive to the therapy, whereas tumour resection histopathologic analysis showed the destruction of tumour vessels and the elimination of tumour cells.

Inhibitors of PDGFR, FGF-2 and MMPs need to be developed in light of what we report in this review [115]. One possible strategy may involve the use of statins to reduce the expression of FGF-2 as demonstrated by Tsubaki in a mouse osteosarcoma model [116]. The use of Suramin for non-small cells lung cancer and breast cancer as an inhibitor of different growth factors such as FGF, VEGF, PDGF open the possibility for the use of this drugs in STS [117]. The use of synthetic peptides derived from TIMP2 to inhibit critical MMPs as well as endothelial cell growth by binding to IGF-1R and/or integrin **α**3**β**1 are two other possible strategies [118]. In a phase I/II trial, the use of batimastat, one of the first generation of metalloproteinase inhibitors was evaluated in patients with malignant ascites. A response to treatment was seen in about half the evaluable patients with advanced malignant disease [120]. Actually, 57 study of anti-metalloproteinases inhibitors are registered as clinical trial (www.clinicaltrials.gov) for different type of tumours.

**Figure 1:**
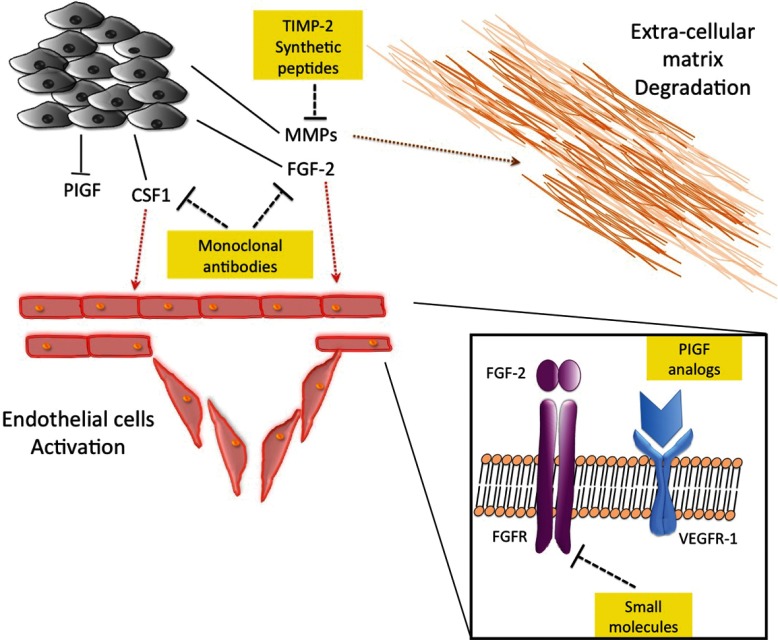
New angiogenic targets in STS New possible angiogenic targets in STS are represented. The yellow boxes indicate the targets for new drug development for STS. TIMP2- synthetic peptides could block or retard extra-cellular matrix degradation, a crucial step in tumour cells migration. Monoclonal antibodies against FGF2 and CSF-1 could interfere with the endothelial cells activation and the consequent new-angiogenesis, a crucial step for tumour mass survival and metastatization. The use of small molecule directed to the FGF2-receptor could have an analogue effect. PIGF analogues could normalize the tumour vessel blocking tumour cells extravasation.

When considering novel therapeutic targets, some other proteins need to be mentioned. In non-gynecological leiomyosarcoma, macrophage CSF1 (colony-stimulating factor 1) showed a stronger correlation with tumour vascularization than VEGF, resulting in a possible candidate for target therapy [121]. In a murine model of fibrosarcoma, PlGF (placental growth factor), a ligand of VEGFR-1, showed important effects on vascular remodelling and normalization, altering tumour growth. Thus, modulation of PlGF concentration or PlGF analogue synthesis could be used as a new therapeutic approach [58]. A representation of possible new targets is schematized in Figure 1.

## DISCUSSION AND CONCLUSIONS

Only a few studies concerning STS focus on tumour neovascularization and proangiogenic factors and look for a correlation with the patients prognosis/survival. These studies demonstrate that intratumoural MVD may not accurately represent the angiogenic capacity of STS. On the other hand, the importance of neoangiogenesis in soft tissue tumours is confirmed by the arising number of publications comparing angiogenesis mediators with clinical features of patients with STS. The efficacy of anti-angiogenic therapies in other types of cancer is well documented. Some *in vivo* studies open the way for the effective use of anti-angiogenic agents against STS. In athymic nude mice injected with an MFH (malignant fibrous histiocytoma) cell line, the treatment with bevacizumab suppressed MFH tumour growth by inhibiting tumoural angiogenesis [122]. In a genetically engineered mouse model of soft tissue sarcoma, the use of TKI sunitinib inhibited STS growth by affecting both tumour vasculature and cancer cells [123]. This permits to hypothesize that the STS cells may express some of the target of the TKIs even if there are only limited evidence at the present. An increasing number of anti-angiogenic agents are under investigation for STS, primarily in phase I/II trials (see Table 1). These studies demonstrate a limited toxicity and a partial response rate but remains the necessity to perform additional investigations. An important possibility from this point of view is the application of a combination therapy as demonstrated by some clinical trials (see Table 2). In most of combinatorial trials, the results seem to be better than the one obtaining from a single agent treatment, but it remains unclear the real role of the anti angiogenic to the tumour progression. Thus the question if STS are suitable for the target therapy is still unanswered. More recently the question of selective sensitivity of sarcoma subtypes emerged. So one can state, in general, that using unselected populations in soft tissue sarcoma trials increases the number of patients recruited, but at the same time diminishes the chance of reaching a statistically significant clinical benefit. Because of the rarity of STS, the subsetting creates a challenge for future trial design and for a real target therapy. Indeed the better understanding of the angiogenic asset in this often mortal condition prompt the exploration of new anti-tumour compounds targeting this pathway in a specific way.
